# Human Platelets Contain, Translate, and Secrete Azurocidin; A Novel Effect on Hemostasis

**DOI:** 10.3390/ijms23105667

**Published:** 2022-05-18

**Authors:** Alba Soledad Aquino-Domínguez, Víctor Acevedo-Sánchez, Diego Sait Cruz-Hernández, Saraí Remedios Sánchez-Aparicio, María de los Ángeles Romero-Tlalolini, Rafael Baltiérrez-Hoyos, Luis Manuel Sánchez-Navarro, Honorio Torres-Aguilar, José Bustos-Arriaga, Sergio Roberto Aguilar-Ruiz

**Affiliations:** 1 Departamento de Biomedicina Experimental, Facultad de Medicina y Cirugía, Universidad Autónoma “Benito Juárez” de Oaxaca, Oaxaca 68120, Mexico; albasoledadaquinod@outlook.es (A.S.A.-D.); victor_acevedo_schz@hotmail.com (V.A.-S.); cruzdiegos@hotmail.com (D.S.C.-H.); sydney_0596@hotmail.com (S.R.S.-A.); sluismanuel81@hotmail.com (L.M.S.-N.); 2 Consejo Nacional de Ciencia y Tecnología, Facultad de Medicina y Cirugía de la Universidad Autónoma Benito Juárez de Oaxaca, Oaxaca 68120, Mexico; mdlaromerotl@conacyt.mx (M.d.l.Á.R.-T.); rbaltierrezho@conacyt.mx (R.B.-H.); 3 Facultad de Ciencias Químicas, Universidad Autónoma “Benito Juárez de Oaxaca”, Oaxaca 68120, Mexico; qbhonorio@hotmail.com; 4 Laboratorio de Biología Molecular e Inmunología de Arbovirus, Facultad de Estudios Superiores Iztacala, Universidad Nacional Autónoma de México, Tlalnepantla, Estado de México 54090, Mexico; jose.bustos@iztacala.unam.mx

**Keywords:** platelets, azurocidin, megakaryoblasts, translation, platelet aggregation, extracellular vesicles

## Abstract

Platelets play a significant role in hemostasis and perform essential immune functions, evidenced by the extensive repertoire of antimicrobial molecules. Currently, there is no clear description of the presence of azurocidin in human platelets. Azurocidin is a 37 kDa cationic protein abundant in neutrophils, with microbicidal, opsonizing, and vascular permeability-inducing activity. Therefore, this work aimed to characterize the content, secretion, translation, and functions of azurocidin in platelets. Our results show the presence of azurocidin mRNA and protein in α-granules of platelet and megakaryoblasts, and stimulation with thrombin, ADP, and LPS leads to the secretion of free azurocidin as well as within extracellular vesicles. In addition, platelets can translate azurocidin in a basal or thrombin-induced manner. Finally, we found that the addition of low concentrations of azurocidin prevents platelet aggregation and activation. In conclusion, we demonstrate that platelets contain, secrete, and translate azurocidin, and this protein may have important implications for hemostasis.

## 1. Introduction

Platelets are small, disk-shaped, anucleated cells and the most abundant in blood after erythrocytes. The main structural elements are the plasma membrane, the open cannular system (OCS), a dense tubular system (DTS), a spectrin-based membrane skeleton, and an actin-based cytoskeleton network. These cells also present a peripheral band of microtubules and various organelles, such as α-granule, dense granules, peroxisomes, lysosomes, and mitochondria. Moreover, several recent studies have reported that they exert a pivotal role in several processes, from primary hemostasis to innate immunity [1]. Once released from their megakaryocytic precursors located in bone marrow, platelets enter the bloodstream and circulate for 8 to 10 days. Despite lacking a nucleus, platelets contain stable messenger RNA transcripts (mRNAs) and the translation machinery for protein synthesis inherited from their cellular precursors [1,2,3].

The primary described function of platelets is in hemostasis; in this process, platelets detect vascular damage by recognizing components of the subendothelium, such as collagen, von Willebrand factor, and extracellular matrix proteins, through glycoproteins present on their surface. Platelet activation forms a hemostatic plug caused by their aggregation and fibrin deposits [4,5]. As part of their participation in the innate immune response, platelets contain molecules with antimicrobial activity, such as kinocidins; CXCL4, CXCL7, and CCL5, and cationic host defense peptides (CHDPs); human neutrophil peptide (HNP) 1, human beta-defensins (HBD) 1–3, and cathelicidin LL-37. Different stimuli such as lipopolysaccharide (LPS), Adenosine diphosphate (ADP), and mainly thrombin induce the secretion of microbicidal molecules from platelets [6]. A clear example of a platelet molecule with microbicidal action is CXCL4 (PF4, platelet factor 4), a very abundant protein in platelet granule-α and one of its main secretion products; CXCL4 has a broad spectrum of antimicrobial action, including on bacteria as *E. coli*, *S. aureus*, and *S. typhimurium*; fungus as *C. albicans*, as well as the antiviral response against human immunodeficiency virus (HIV) and influenza A virus (IAV) [6,7]. In addition to the above, it has been described that those molecules with antimicrobial activity can induce [8,9] or inhibit [10] platelet aggregation, increasing the evidence of the functional link between hemostasis and platelet immune response.

Despite recent advances in the description of antimicrobial molecules in platelets, their totality is not yet known, and such is the case of azurocidin (AZU1) or HBP (heparin-binding protein), a 37 kDa protein, so-called because of its localization and abundance in the azurophilic and secretory granules of neutrophils [11,12]. Azurocidin is vital in inducing edema and vascular permeability; likewise, azurocidin is microbicidal, opsonizing, chemotactic, and activates monocytes and macrophages [13,14,15,16]. Serial analysis of gene expression (SAGE) has reported the presence of the mRNA for azurocidin in human umbilical cord cell-derived megakaryoblasts [17] and the protein in ADP-activated human platelet-derived extracellular vesicles (pEVs) from the proteomic analysis [18]. However, information about azurocidin in human platelets is still incomplete. Therefore, this research aimed to demonstrate the presence, location, secretion, translation, and functions of azurocidin in human platelets.

## 2. Results

### 2.1. Platelets and Megakaryoblasts Contain Azurocidin mRNA and Protein

**Figure 1:**
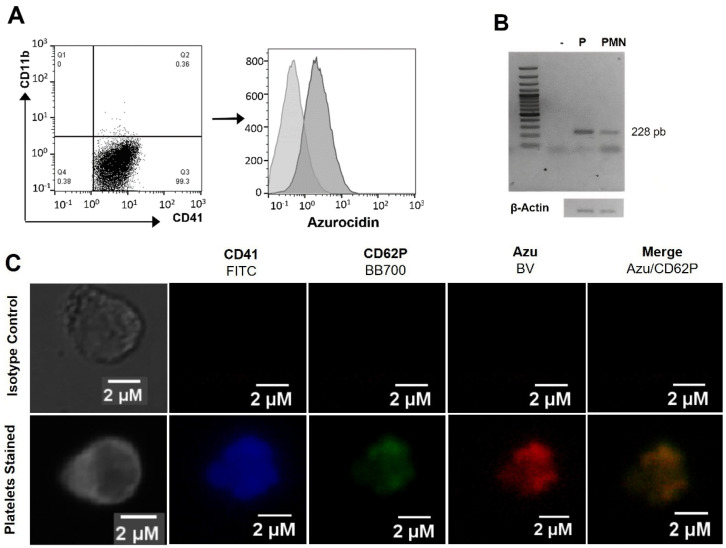
Human platelets express azurocidin mRNA, and the protein is localized in the α−granules. (**A**) flow cytometry analysis of platelets purified from peripheral blood of healthy donors. Surface staining with CD41 was used as a lineage marker and CD11b to exclude leukocyte contamination. Azurocidin expression comes from a prior selection of the CD41^+^ population, and the dark gray histogram represents intracellular staining with a mAb specific for azurocidin; the light gray histogram indicates staining with the isotype control mAb. (**B**) RT-PCR amplicons of azurocidin in platelets (P) and polymorphonuclear leukocytes (PMN) resolved on 2% agarose gels, and β-actin expression was used as housekeeping control. Both (**A**,**B**) data are representative of five independent experiments (**C**). Confocal microscopy of purified platelets stained intracellularly with mAbs against CD41 (blue), CD62P (green), and azurocidin (red). The scale bar equals 2 µm (Representative images of three independent assays).

Human platelets were purified as indicated in the materials and methods, and purity was assessed by microscopy and flow cytometry using a mAb directed against CD41, which defines platelet lineage. We used the mAb directed against CD11b (present on leukocytes) to determine the degree of purity. Figure 1A shows that CD41^+^ platelets lack the presence of contaminating leukocytes and that these cells contain intracellular azurocidin. Following RNA extraction and using RT-PCR, the presence of the azurocidin transcript was identified (Figure 1B). Finally, using mAbs directed against CD41, CD62P (stored in the α-granules), and azurocidin, by confocal microscopy, azurocidin was shown to localize to the α-granules of platelets (Figure 1C).

**Figure 2:**
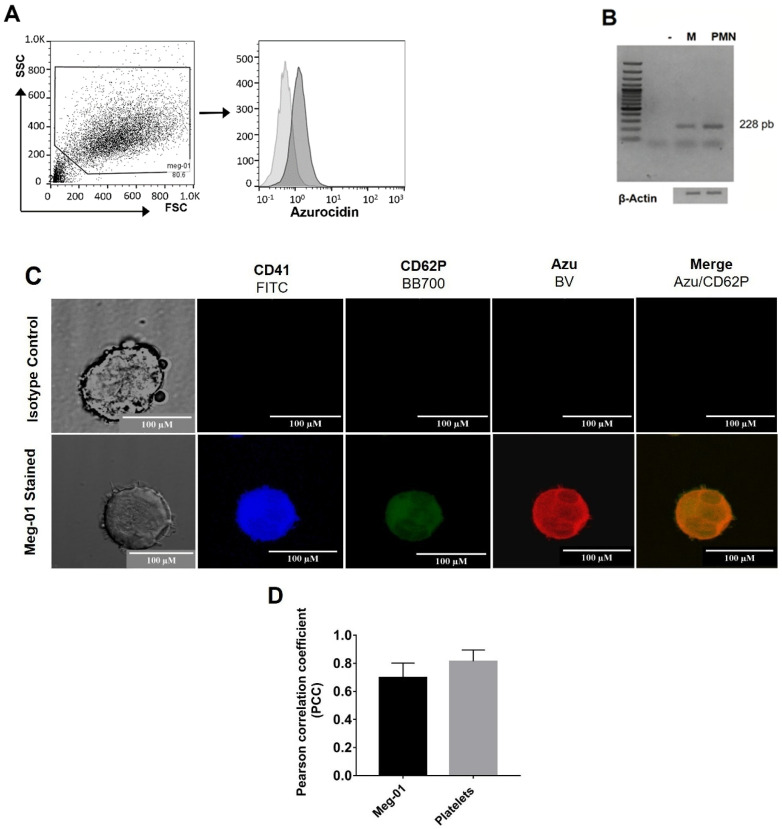
Meg−01 cells express azurocidin. (**A**) Intracellular staining for azurocidin was performed in the Meg-01 cell line using a specific mAb and analyzed by flow cytometry. In the figure, it is depicted in a dark gray histogram, while the light gray histogram indicates staining with the isotype control mAb. (**B**) Total RNA from Meg-01 cell cultures was extracted and analyzed for azurocidin expression by RT-PCR, amplicons of the expected size (228 bp) from cell line (M) and peripheral blood polymorphonuclear leukocytes (PMN) were resolved on 2% agarose gels, β−actin expression was used as housekeeping control. Both (**A**,**B**) data are representative of five independent experiments. (**C**) Confocal microscopy of Meg-01 cells, stained intracellularly with mAbs against CD41 (blue), CD62P (green), and azurocidin (red). Scale bar equals 100 µm (representative images of 3 independent assays). (**D**) The graphs show the averages ± SD of the Pearson correlation coefficient (PCC) value obtained for each image after background correction in the Regions of Interest (ROI) in five selected areas per slice of the co-localization visualized between CD62P and Azurocidin from 3 independent assays and analyzed with the Coloc-2 co-localization plug-in of FIJI.

Like platelets, megakaryoblasts contain azurocidin in alpha granules (Figure 2C). Using the same experimental tools, we found mRNA (Figure 2A) and azurocidin protein (Figure 2B) in megakaryoblasts. Co-localization analysis between CD62P and azurocidin indicates that Meg-01 cells have a correlation value of 0.698 ± 0.1, while platelets of 0.813 ± 0.08, considering that the maximum value of the correlation is 1.0. These results indicate that azurocidin is mainly stored in the α-granules of platelets (Figure 2D).

### 2.2. Platelets and Megakaryoblasts Secrete Azurocidin Free and within EVs

**Figure 3:**
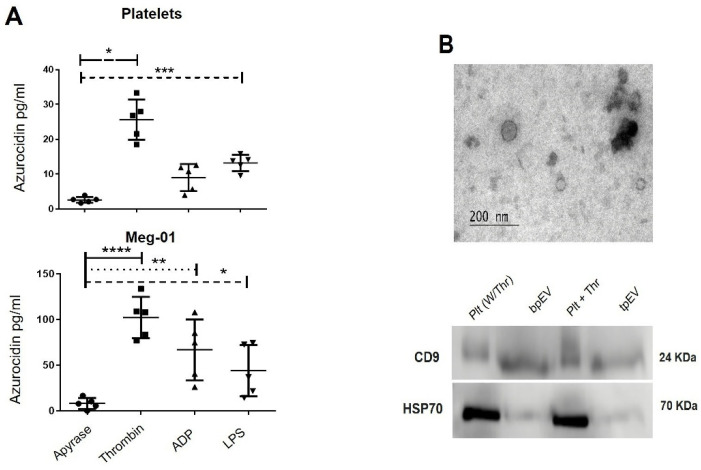

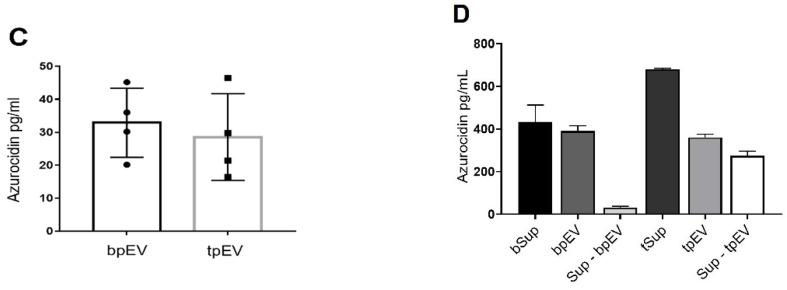
Platelets secrete azurocidin free and within EVs. (**A**) Stimulated 1 ×10^7^ peripheral blood platelets (top) and 1 × 10^6^ Meg-01 cells (bottom) with 1 U thrombin, 5 µM ADP, 1µg LPS, or 0.25 U/mL Apyrase at 37 °C for 30 min, and azurocidin secretion was quantified by ELISA. (**B**) 2 × 10^9^ platelets were treated with 1 U thrombin, or in the absence of thrombin afterward, platelet-derived extracellular vesicles (pEVs) were obtained as indicated in materials and methods (Section 4.7) and characterized by transmission electron microscopy (TEM), a representative micrograph of pEVs is shown in the figure (top). Moreover, the proteins were used to perform a Western blot in search of characteristic markers of EVs, present in platelets treated with thrombin (Plt + Thr) or untreated (Plt W/Thr) and in EVs derived from platelets under basal conditions (bpEVs) or treated with thrombin (tpEVs). (**C**) Proteins derived from bpEVs and tpEVs were used to detect the presence of azurocidin by ELISA. (**D**) The supernatant of 2 × 10^9^ thrombin-treated platelets (tSup) for 30 min, or without treatment (bSup), from which bpEVs and tpEVs were extracted by ultracentrifugation, as well as supernatants without pEVs (Sup-bpEV and Sup-tpEV) were obtained. Azurocidin concentration was measured by ELISA in all conditions. The graphs represent the mean ± standard deviation of 5 (**A**), 4 (**C**), or 2 (**D**) independent experiments in the figure. Statistical significance was determined by Dunn Sidak multiple comparison test and is represented as * *p* < 0.05, ** *p* < 0.01, *** *p* < 0.001, **** *p* < 0.0001.

Considering the found localization of azurocidin in the α-granule of platelets and Meg-01 cells, the ability of these cells to secrete this protein with stimuli that trigger granule secretion, such as thrombin, ADP, and lipopolysaccharide, to benefit its baseline condition of non-activation an apyrase treatment condition was used, and subsequently evaluated by ELISA. Our results show that both cell types can secrete azurocidin in response to stimulation, exhibiting different values according to the agonist employed (Figure 3A). Azurocidin secretion by both cell types was significantly higher in the presence of thrombin.

On the other hand, platelets are known to be one of the primary sources of EVs in blood [19,20], and the presence of azurocidin in pEVs has been reported in proteomic analyses [18]. Therefore, we decided to test this finding by purifying pEVs derived from thrombin treatment (tpEVs) or basal conditions (bpEVs) for 30 min. Figure 3B (top) shows a micrograph obtained by transmission electron microscopy (TEM) of the pEVs obtained and the characteristic CD9 and HSP90 markers of EVs (Figure 3B, bottom). The proteins obtained from the pEVs were used to determine the presence of azurocidin by ELISA. The results show that bpEVs and tpEVs contain similar azurocidin concentrations (32.8 ± 10.5 pg/mL vs. 28.5 ± 13.4 pg/mL) (Figure 3C), demonstrating that platelets release azurocidin-containing EVs under both basal and thrombin-activated conditions.

To determine the amount of azurocidin free or within pEVs, the total supernatant obtained from platelets stimulated with thrombin (tSup) or without stimulation (bSup) for 30 min was divided into two parts. One to obtain EVs by ultracentrifugation, and the other to obtain EVs-depleted supernatants. Azurocidin concentration was measured in EVs, and EVs-depleted supernatants separately. The results indicated that azurocidin is basally released from platelets (432.4 ± 80.53 pg/mL), but stimulation with thrombin enhances azurocidin secretion (679.7 ± 4.8 pg/mL). Interestingly, azurocidin concentration in bSup was comparable to that in bpEVs (432.4 ± 80.53 vs. 390.4 ± 15.2 pg/mL), and a low concentration was detected in bpEVs-depleted bSup (Sup-bpEVs) (26.94 ± 5.4 pg/mL), suggesting that most of the azuricidin secreted by platelets under basal conditions are found in EVs. The azurocidin concentration in the supernatant of thrombin-stimulated platelets was distributed in tpEVs (360.7 ± 14.7 pg/mL) and in the supernatant depleted from pEVs (Sup-tpEVs) (274.1 ± 21.53 pg/mL), suggesting that thrombin stimulation leads to the secretion of free azurocidin as in tpEVs (Figure 3D). In conclusion, our results indicate that human platelets secrete free azurocidin as in EVs.

### 2.3. Platelets Translate Azurocidin

**Figure 4:**
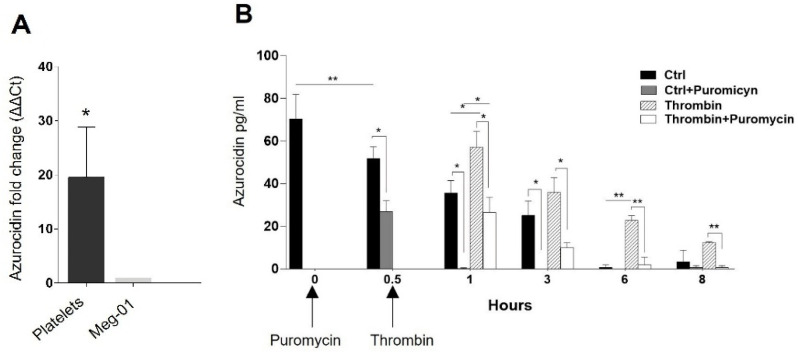
Platelets translate azurocidin. (**A**) Total RNA from Meg-01 cell line and peripheral blood platelets was extracted to quantify azurocidin expression by qRT-PCR. Graphs show the average expression values, calculated using the 2−ΔΔCt method and normalized for β-actin expression. Data are representative of three independent experiments. (**B**) 50 × 10^6^ washed platelets from peripheral blood were treated with puromycin, and 0.5 h later received thrombin stimulation (the arrow indicates the time at which the treatments were added). Platelets were not stimulated with thrombin in the control condition, as indicated in the figure. Subsequently, we obtained total protein (cell and supernatant) at different times and quantified the amount of azurocidin by ELISA. In the figure, the graphs represent the mean ± standard deviation of 3 (**A**) or 4 (**B**) independent experiments. Statistical significance was determined by Dunn Sidak multiple comparison test (**A**) and 2-way ANOVA with repeated measures (**B**) and is represented as * *p* < 0.05, ** *p* < 0.01.

**Figure 5:**
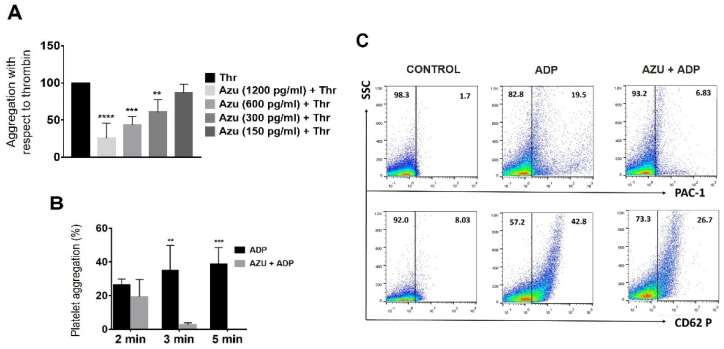
Inhibition of platelet aggregation and activation mediated by azurocidin. (**A**) Purified platelets were stimulated with 2 U of thrombin or pretreated (30 s before) with different concentrations of azurocidin, and platelet aggregation was subsequently evaluated. The figure shows the percentage of aggregation concerning thrombin treatment (100%). (**B**) The figure represents platelet aggregation over time of platelet-rich plasma (PRP) treated with 2 µM ADP or with the addition of 1200 pg/mL azurocidin. (**A**,**B**) represent the mean ± standard deviation of 4 independent experiments. Statistical significance was determined by a Dunnett’s (**A**) and Sidak’s (**B**) multiple comparison test, and is represented as ** *p* < 0.01, *** *p* < 0.001, **** *p* < 0.0001. (**C**) Representative dot plots of 3 independent experiments for surface expression of PAC-1 (upper) and CD62P (lower) in platelets that received the treatments indicated in the figure. The tests are the result of a previous selection of CD41^+^ platelets.

Subsequently, we compared the amount of azurocidin mRNA in platelets and Meg-01 cells by qRT-PCR and found that platelets have, on average, 20-fold more of this transcript by normalizing its expression with the actin gene (Figure 4A). Therefore, we wondered whether peripheral blood platelets require protein translation to produce azurocidin. For this purpose, unstimulated or thrombin-stimulated platelets were treated with the translation inhibitor puromycin, and the total amount of azurocidin (secreted and soluble) was assessed at 0, 0.5, 1, 3, 6, and 8 h by ELISA. The results show that the amount of azurocidin decreases from 0.5 h, and most of its maintenance depends on constitutive translation observed from 0.5 h onwards (Figure 4B). Interestingly, treatment with thrombin significantly increases the amount of azurocidin concerning the basal condition after 0.5 h of thrombin administration and maintains this effect subsequently, indicating a rapid and inducible translation by this agonist since treatment with puromycin decreases this activity (Figure 5B). The results obtained lead us to conclude that platelets translate azurocidin constitutive and inducibly at early times by thrombin stimulation. As part of these assays, platelet viability was assessed under all conditions and at all time points by incorporating trypan blue (data not shown).

### 2.4. Azurocidin Inhibits Platelet Aggregation and Activation

As mentioned earlier, some microbicidal molecules present in platelets can modulate platelet activation. For this reason, we evaluated the effect of azurocidin on this biological function. Our findings show that different concentrations of azurocidin (150–1200 pg/mL) do not induce aggregation of purified platelets (data not shown). However, when pretreated (30 s before) with these same concentrations and stimulated with thrombin, concentration-dependent platelet aggregation is significantly inhibited (Figure 5A). Similarly, pretreatment with azurocidin (1200 pg/mL) inhibits aggregation of ADP-stimulated platelet-rich plasma (PRP) over time, with the most significant effect observed at 5 min (Figure 5B). Finally, we found that azurocidin also inhibits the expression of platelet activation molecules CD62P and PAC-1 (Figure 5C). The above results suggest a new role of azurocidin in regulating hemostasis.

## 3. Discussion

Platelets are the smallest circulating cells primarily involved in hemostasis but have gained interest due to their participation in divergent functions, including the immune response. In innate host defense, platelets produce antimicrobial and chemoattractant mediators [6,21]. Nevertheless, the presence of azurocidin protein in platelets and megakaryoblasts had not been fully characterized.

Our results confirm the presence of azurocidin mRNA in megakaryoblastic and peripheral blood platelets, suggesting that platelets could acquire this transcript from their megakaryocyte precursor [22]. We subsequently confirmed the expression of azurocidin protein, within the α-granule, of both platelets and Meg-01 cells, in the latter also in an extra-granular manner. In addition to azurocidin, it is now known that α-granules contain a variety of molecules with microbicidal activity, such as Basic Platelet Protein (PBP), CXCL4, CCL5, and HNP-1 (defensin-α1) [6]. Recently, it has been shown that megakaryocytes contain and transfer defensin-α1 to nascent platelets, although they can also acquire it from plasma [23]. It would be relevant to study whether this acquisition system also occurs for platelet azurocidin.

After knowing the presence of azurocidin in the α-granules of platelets and megakaryoblasts, we demonstrated that the stimuli leading to degranulation (thrombin, LPS, and ADP) lead to azurocidin secretion, at a time of 30 min, in addition, we corroborated the presence of azurocidin inside and apparently also bonded at the surface de las tpEVs and bpEVs. Our previous demonstrations open new possibilities to investigate the microbicidal function of pEVs, as has been described for neutrophil-derived EVs, which are rich in MPO (myeloperoxidase) and lactoferrin being able to inhibit the growth of *S. aureus* [24]. On the other hand, EVs derived from neutrophils stimulated with *A. fumigatus* increase their azurocidin content, and the expression of this molecule from transfection inside *A. fumigatus* reduces their growth [25]. However, the function that platelet azurocidin might perform is still unknown.

In addition to releasing azurocidin, our results show that platelets constitutively translate this protein, such that after 1 h, the azurocidin produced by platelets derives entirely from translation. Contraction of basal translation has also been reported to maintain plasminogen activator inhibitor (PAI-1), reaching its highest production in platelets at 3 h in vitro [26]. Similarly, other abundant platelet proteins such as myosin, actin, glycoproteins (GP) Ib, IIb/IIIa, fibrinogen, and thrombospondin also show constitutive de novo synthesis [27,28]. On the other hand, we also found that platelets can perform inducible translation of azurocidin, reaching its highest concentration at 30 min after thrombin stimulation, which is interesting because reports on activation-inducible translation in platelets have shown increased synthesis at 6 h for PAI-1 [26], and 18 h for IL-1β [29] and proteins that regulate cytoskeleton and motility [30]. We do not know the mechanism of this finding, and we suppose that some platelet mRNAs are translated at a different rate from each other, probably some more rapidly, which could depend on their access to ribosomes and the subsequent formation of polysomes [29,30]. The physiological impact of azurocidin translation in peripheral blood platelets is still unknown, but it is known that the treatment of platelets with translation inhibitors affects their ability to aggregate [31].

On the other hand, we found that azurocidin inhibits the induced aggregation of both washed platelets and platelets in PRP in a concentration-dependent manner and reduces the expression of the activation proteins CD62P and PAC-1. It has been previously published those other molecules with microbicidal activity, HNP-1, and LL-37, induce platelet aggregation by binding to their receptors on these cells, which correspond to GP IIb/IIIa and FPR2/ALX, respectively [8,9]. Nevertheless, the treatment of platelets with lactoferrin or fragments derived from it inhibits ADP-induced platelet aggregation due to recognition of the lactoferrin receptor, a 105 KDa protein [10,32]. The azurocidin receptor is so far unknown [13], although an interaction between azurocidin with CR3 (CD11b/CD18) may occur [33]. Other authors have shown that the effects of azurocidin on vascular permeability depend on its cationic surface rather than receptor recognition [16]. Therefore, it is essential to determine how azurocidin mediates its inhibitory effects on platelets.

## 4. Materials and Methods

### 4.1. Purification of Platelets from Peripheral Blood

The blood obtained for the development of this study came from clinically healthy donors, without the use of aspirin or drugs that could affect platelet function, at least two weeks before sample collection. Blood was collected from the antecubital vein using Vacutainer tubes with ACD (BD Biosciences, Franklin Lakes, NJ, USA). PRP is obtained by centrifuging the blood at 110× *g* for 15 min. The platelet purification was performed using CGS-EDTA buffer (1.29 mM sodium citrate, 3.12 mM citric acid, 4.99 mM ethylenediaminetetraacetic acid, pH 6.5) at a 1:1 ratio (*v*/*v*), and centrifuged at 110× *g* for 15 min. Subsequently, the supernatant was centrifuged at 480× *g* (TX-150 rotor, radius; 144 mm, Thermo Fisher Scientific, Waltham, MA, USA) for 15 min, and the pellet obtained was washed twice with GSG-EDTA, centrifuging at 480× *g*; the first wash was 15 min, and the second wash was 8 min. The purity of the platelets was verified by light microscopy and flow cytometry, the latter using the monoclonal antibodies (mAb) APC mouse anti-human CD11b (clone: D12) and FITC mouse anti-human CD41 (clone: HIP8), and the isotype controls APC mouse IgG2a, κ isotype control (clone: G155-178), and FITC mouse IgG3, κ isotype control (clone: J606), all from BD Biosciences. Finally, platelets were fixed with 4% paraformaldehyde for 30 min and acquired in a MACSQuant flow cytometer (Milteny Biotec, Bergisch Gladbach, Germany). The obtained data were analyzed using FlowJo software Version 10 (FlowJo, Ashland, OR, USA).

### 4.2. Meg-01 Cell Line Culture

The megakaryoblastic cell line MEG-01 (CLR-2021 TM) (ATCC, Manassas, VA, USA) was kept alive and proliferating in RPMI 1640 medium (ATCC). RPMI medium was supplemented with L-glutamine and 10% fetal bovine serum (FBS) (Sigma-Aldrich, St. Louis, MO, USA) under 37 °C and 5% CO_2_ culture conditions.

### 4.3. Azurocidin mRNA Expression by RT-PCR and qRT-PCR

Total RNA was extracted from platelets and MEG-01 cells using the RNeasy Mini Handbook kit (Qiagen, Hilden, Germany) and treated with the DNA-free DNA removal kit (Invitrogen, Waltham, MA, USA). Reverse transcription of the RNA into cDNA was using the RevertAid First Strand cDNA Synthesis Kit (Thermo Scientific, MA, USA). Subsequently, the PCR assay was carried out with the Phusion Hot Start II High-Fidelity DNA Polymerase (Thermo Scientific, MA, USA), azurocidin-specific primers (sense 5′-TCAGAATCAAGGCAGGCACT-3′) (antisense 5′-TGAAGCAGCATCAGGTCGTT-3′), and primmer (sense 5′-GCGTTACACCCTTTCTTGAC-3′) and (antisense 5′-TTGTGAACTTTGGGGGATGC-3′) for β-actin were used as control. Amplification products were resolved on a 2% (*w*/*v*) agarose gel and visualized by ethidium bromide staining. The identity of the products was confirmed through Sanger sequencing. The qRT-PCR assay was carried out using the Maxima SYBR Green/ROX qPCR Master Mix (2X) (Thermo Scientific, MA, USA) on a Step One Plus Real-Time equipment (Applied Biosystems, Foster City, CA, USA). Results were calculated using the formula 2−ΔΔCt, and β-actin was used for normalization.

### 4.4. Identification of Azurocidin by Flow Cytometry in Platelets and Megakaryoblasts

Meg-01 cells and human peripheral blood platelets were collected and washed with Tyrodes buffer and permeabilized with the Cytofix/Cytoperm kit (BD Biosciences). Subsequently, the cells were treated with a primary human anti-azurocidin mouse mAb (clone: 246,322) or the mouse IgG1 isotype control mAb (clone: 11,711) (both from R&D Systems, Minneapolis, MN, USA) and then with a polyclonal goat anti-mouse-IgG antibody (Brillant Violet 421, BioLegend, San Diego, CA, USA). We used FITC mouse anti-human CD41 and APC mouse anti-human CD11b mAbs or their respective isotype controls (indicated in Section 4.1) upon completion. Finally, the cells were fixed and analyzed by flow cytometry (Section 4.1).

### 4.5. Localization of Azurocidin in Platelets and Meg-01 Cells by Immunofluorescence

Freshly purified platelets or Meg-01 cells were suspended in modified Tyrode’s buffer, placed on histological slides, and fixed with IC fixation buffer (Thermo Fisher Scientific, USA) at 4 °C for 30 min. Then the cells were treated with a blocking solution (1% BSA + 0.1% Tween 20 in 1× permeabilization buffer) (Thermo Fisher Scientific, USA). Cells on slides were incubated with the anti-human azurocidin mAb or its isotype control (indicated in Section 2.4) at 4 °C for 12 h and then treated with goat anti-mouse-IgG secondary antibody (Brillant Violet 421, USA) for 1 h at room temperature. Subsequently, the sample was incubated with the mAbs FITC mouse anti-human CD41 or its isotype control (Section 2.4) and BB700 Mouse anti-human CD62P (Clone: AK-4, BD Biosciences), or BB700 mouse IgG1, κ isotype control (clone: X40, BD Biosciences). Finally, the samples were imaged on the LSM 5 Pascal confocal microscope (Carl Zeiss: Oberkochen, Germany), using a 100× objective, and obtained analyzed images were with the Leica Application Suite X software (LAS-X, Leica-Microsystems, Wetzlar, Hesse-Darmstadt, Germany).

Co-localization analysis was performed on each image after background correction in the Regions of Interest (ROI) in five selected areas per slice of the co-localization visualized between CD62P and Azurocidin in each of the three independent experiments and analyzed with the Coloc-2 co-localization complement of FIJI. The results were represented as the average ± standard deviation of the Pearson correlation coefficient (PCC).

### 4.6. Azurocidin Secretion Assay

Meg-01 cells (1 × 10^6^) and platelets (1 × 10^7^) were collected and stimulated with 1 unit of thrombin (Dade Behring, Deerfield, IL, USA), 5 μM of ADP (Sigma-Aldrich), or 1 μg of LPS (Sigma-Aldrich), and apyrase was used to benefit its baseline condition of non-activation. (0.25 U/mL) (Sigma-Aldrich) was used, and all stimuli were incubated at 37 °C for 30 min. The supernatant was collected, and the secreted azurocidin was quantified by ELISA using an optical density. Readings were performed at 450 nm using the Chromate 4300 microplate reader (Awareness Technology Inc.; Palm City, FL, USA) with the Leica Suite 345 X software application (LAS-X, Leica-Microsystems).

### 4.7. Identification of Azurocidin in EVs

Platelets obtained as previously described (Section 4.1) and 2 × 10^9^ were treated with 1 U thrombin, or in the absence of thrombin (control condition), for 30 min, at 37 °C. Subsequently, platelets were removed by centrifugation at 100× *g* for 10 min, followed by 650× *g* for 5 min at room temperature. The pellets obtained were removed and discarded after each centrifugation step, and the supernatants were employed to obtain the extracellular vesicles. According to the manufacturer’s instructions, extracellular vesicles were isolated using the miRCURY Exosome Isolation Kit (Qiagen, Germany). Briefly, the samples were centrifuged at 3200× *g* for 15 min at room temperature to remove cell debris. The supernatants were transferred to a new tube and added precipitation buffer B. Then, samples were vortexed and incubated at 4 °C overnight. After the incubation, all the samples were centrifuged at 10,000× *g* for 30 min at 20 °C and discarded the supernatant. The pellets of EV were resuspended in PBS or RIPA buffer. Employed fixed EV in PBS with 2% glutaraldehyde for transmission electron microscopy and those in RIPA buffer (Santa Cruz Biotechnology, Santa Cruz, CA, USA) to obtain the proteins. Proteins derived from EVs were used to assess azurocidin concentration by ELISA (Section 4.6). Additionally, to evaluate in more detail the secretion of azurocidin, the supernatant of activated platelets was subject to ultracentrifugation to get EV. The supernatant was centrifuged at 2000× *g* for 20 min twice and the pellets were discarded. The supernatant was centrifuged at 95,000× *g* for 2 h. The supernatant was separated and employed to ELISA (Section 4.6) and the pellet containing EVs was suspended in PBS and centrifuged at 95,000× *g* for 2 h. The supernatant was discarded and the EVs were treated with RIPA buffer, quantified, and employed for ELISA.

### 4.8. Identification of EVs by Western Blot and TEM

The proteins from platelets and extracellular vesicles were extracted using RIPA buffer (Santa Cruz Biotechnology, USA), and the concentrations were determined by the BCA Protein Assay kit (Thermo Fisher Scientific, USA). Fifteen µg of each sample was loaded on a 10% SDS-PAGE gel, and proteins were transferred to a polyvinylidene difluoride membrane (Thermo Fisher Scientific, USA). The membrane was blocked with 5% non-fat milk in 1× TBST (TBS, 0.05% Tween 20) for 2 h at room temperature and subsequently incubated with primary antibodies anti-CD9 (1:250) (Santa Cruz Biotechnology, USA) and anti-HSP70 (1:1000) (Santa Cruz Biotechnology, USA) overnight at 4 °C. The membranes were washed three times (5 min each time) with TBST and subsequently incubated with the secondary antibody (Jackson ImmunoResearch Laboratories, West Grove, PA, USA) at a dilution of 1:5000 for 2 h at room temperature. Finally, the membranes were washed three times with TBST before scanner the result. The results were scanned on a C-DiGit Blot scanner (LI-COR Biosciences, Lincoln, NE, USA) using Immobilon Crescendo Western HRP Substrate (Millipore, Burlington, MA, USA). Image Studio Digits v.5.2 software (LI-COR Biosciences, USA) was employed for image acquisition. For transmission electron microscopy, the fixed EV were placed onto carbon-coated copper grids to be negatively stained with 2% uranyl acetate. Representative images were obtained with a JEM-1400 transmission electron microscope with an acceleration voltage of 80 kV.

### 4.9. Translation of Azurocidin into Platelets

Peripheral blood platelets were obtained as described in Section 4.1 and plated at a 5 × 10^7^ cells/mL concentration in RPMI medium supplemented with 10% SFB. In some conditions, the platelets received pretreatment with 8 µg/mL puromycin (Sigma-Aldrich), and 30 min later received or did not receive stimulation with 0.5 U of thrombin, platelets without any treatment were used as control. Incubation times were 0.5, 1, 1, 3, 6, and 8 h, at 37 °C and 5% CO_2_. After incubation time, total protein (cellular and secreted) was extracted using RIPA buffer. Finally, azurocidin concentration was determined by ELISA.

### 4.10. Inhibition of Platelet Aggregation and Platelet Activation

Platelet-rich plasma (PRP) was obtained from 8 mL of whole blood anticoagulated with ACD and centrifuged at 100× *g* for 15 min; platelet-poor plasma (PPP) was obtained by centrifugation at 900× *g* for 10 min, and with it, adjusted the number of platelets for the assay to 250 × 10^9^/L, and then their aggregation was evaluated. For thrombin assays, the platelets were purified according to Section 4.1 and adjusted the number of cells to 250 × 10^9^/L with Tyrode’s buffer. Based on the Born method, the Light transmission aggregometry (LTA) assays were performed using a Chrono-Log 500-CA 500CA platelet aggregometer (Havertown, PA, USA). The incubation before performing the assay was 1 min at 37 °C, maintaining the speed of 1200 rpm for 6 min after pretreatment with 15 μL of azurocidin (Azu/CAP37 Protein, Human, Recombinant) (Chesterbrook, PA, USA) with subsequent serial dilutions, the highest concentration being 1200 pg/mL, 0.9% saline solution was used to control before aggregation with ADP (2 μM) in platelets suspended in PPP or Thrombin (2 U) for purified platelets.

On the other hand, 5 × 10^6^ platelets/mL in Tyrode’s buffer were treated with saline solution or 1200 pg/mL Azurocidin for 5 min at 37 °C and 5% CO_2_, then ADP 20 μM or 0.9% saline for an additional 30 min, then the cells were fixed with 1% paraformaldehyde for 30 min and stained with the mouse mAbs PE Cy5 anti-human CD41 (clone: HIP8) (BD Pharmingen, San Diego, CA, USA), FITC anti-human CD62P (clone: AK4) or FITC anti-human PAC-1 (clone: PAC-1) (both from BioLegend, San Diego, CA, USA). The mAbs used as isotype control were PE-Cy5 Mouse IgG1 κ Isotype Control (clone: MOPC-21) (BD Pharmingen) and FITC Mouse IgG1 isotype control, κ Isotype Ctrl (clone: MOPC-21) (BioLegend, San Diego, CA, USA). Finally, the cells were analyzed by flow cytometry (Section 2.1).

## 5. Conclusions

Platelets and megakaryoblasts express azurocidin mRNA and protein, which is contained in their granule-α, and upon stimulation with thrombin, ADP or LPS can secrete it. In addition, platelets can also release azurocidin contained in EV. Moreover, platelets translate azurocidin mRNA both under basal conditions and upon stimulation. Finally, azurocidin inhibits platelet aggregation and platelet activation, which may be a new role of azurocidin in hemostasis.

## Data Availability

Data is contained within the article.
